# Bioprinting in cardiovascular medicine: possibilities, challenges, and future perspectives for low and middle-income countries

**DOI:** 10.1097/JS9.0000000000001537

**Published:** 2024-05-03

**Authors:** Amarveer Malhi, Inderbir Padda, Arun Mahtani, Daniel Fabian, Paul Karroum, Arpita M. Mathews, Tushar Ralhan, Yashendra Sethi, Talha B. Emran

**Affiliations:** a Department of Medicine, CMU School of Medicine, Netherlands, Antilles; b Department of Internal Medicine, Richmond University Medical Center/Mount Sinai, Staten Island, New York, USA; c PearResearch, Dehradun; d Mount Zion Medical College, Kerala; e Department of Medicine, Government Doon Medical College, Dehradun, India; f School of Medicine, St. George’s University, True Blue, Grenada; g Department of Pharmacy, Faculty of Allied Health Sciences, Daffodil International University, Dhaka, Bangladesh

**Keywords:** bioprosthetic, cardiology, low- to middle-income countries, valve replacement

## Abstract

Cardiovascular diseases stemming from various factors significantly impact the quality of life and are prevalent with high mortality rates in both developed and developing countries. In cases where pharmacotherapy proves insufficient and end-stage disease ensues, a heart transplant/surgical repair becomes the only feasible treatment option. However, challenges such as a limited supply of heart donors, complications associated with rejection, and issues related to medication compliance introduce an additional burden to the healthcare system and adversely affect patient outcomes. The emergence of bioprinting has facilitated advancements in creating structures, including ventricles, valves, and blood vessels. Notably, the development of myocardial/cardiac patches through bioprinting has offered a promising avenue for revascularizing, strengthening, and regenerating various cardiovascular structures. Employment loss in developing countries as a circumstance of disability or death can severely impact a family’s well-being and means for sustainable living. Innovations by means of life sustaining treatment options can provide hope for the impoverished and help reduce disability burden on the economy of low- and middle-income countries (LMICs). Such developments can have a significant impact that can last for generations, especially in these countries. In this review, the authors delve into various types of bioprinting techniques, exploring their possibilities, challenges, and potential future applications in treating various end-stage cardiovascular conditions in LMICs.

## Introduction

Highlights

- Cardiovascular diseases significantly impact global quality of life.
- Heart transplants face challenges like limited donors, rejection, and medication issues.
- Bioprinting advancements offer solutions, particularly in creating cardiovascular structures.

Cardiovascular diseases (CVD) lead to a significant number of deaths and disability-adjusted life years (DALYs) globally. In 2019, CVD was responsible for one-third of global deaths: 8.9 million women and 9.8 million men^1^. China, India, Russia, the United States, and Indonesia face the highest burden of CVD mortality^1^. Mortality rates adjusted for age were highest in Uzbekistan, Solomon Island, and Tajikistan^2^. However, in LMICs, primary and secondary prevention approaches are limited due to cost-effective healthcare strategies^2^. A heart transplant is a definitive treatment for various end-stage CVD^3–5^. The scarcity of donor hearts has led to the development of bioprinting, an additive manufacturing technique that can arrange healthy cells, biological molecules, and materials in layers to replicate organs/structures such as ventricles, valves, arteries, and cardiomyocytes^4–6^.

**Figure 1:**
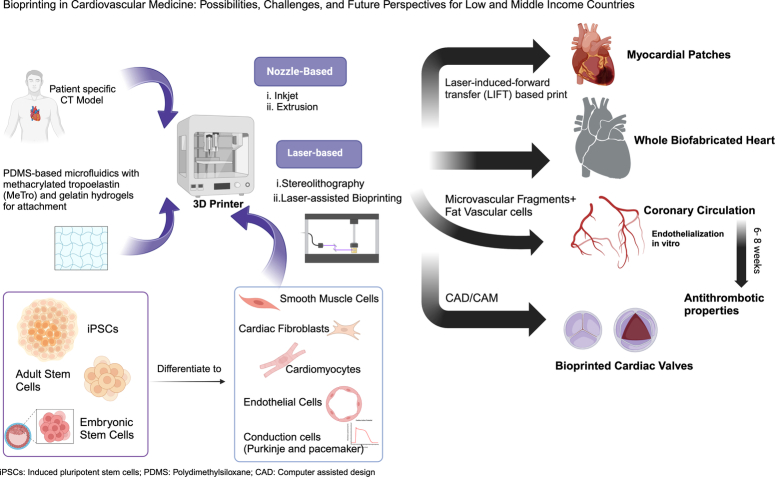
Bioprinting in cardiovascular medicine has facilitated advancements in creating whole biofabricated hearts, myocardial patches, coronary circulation, and cardiac valves. Embryonic stem cells (ESCs), adult stem cells, and induced pluripotent stem cells (iPSCs) differentiate into the cellular population of the heart, which when combined with growth factors promote the induction of myocardium-supportive cell lineages. An in-vivo environment created with microfluidics, which along with patient-specific CT Models yields revolutionary results of 3D bioprinting. CAD, computerassisted design; CAM, computer-aided manufacturing; iPSCs, induced pluripotent stem cells; PDMS, polydimethylsiloxane. Created with BioRender.com.

**Figure 2:**
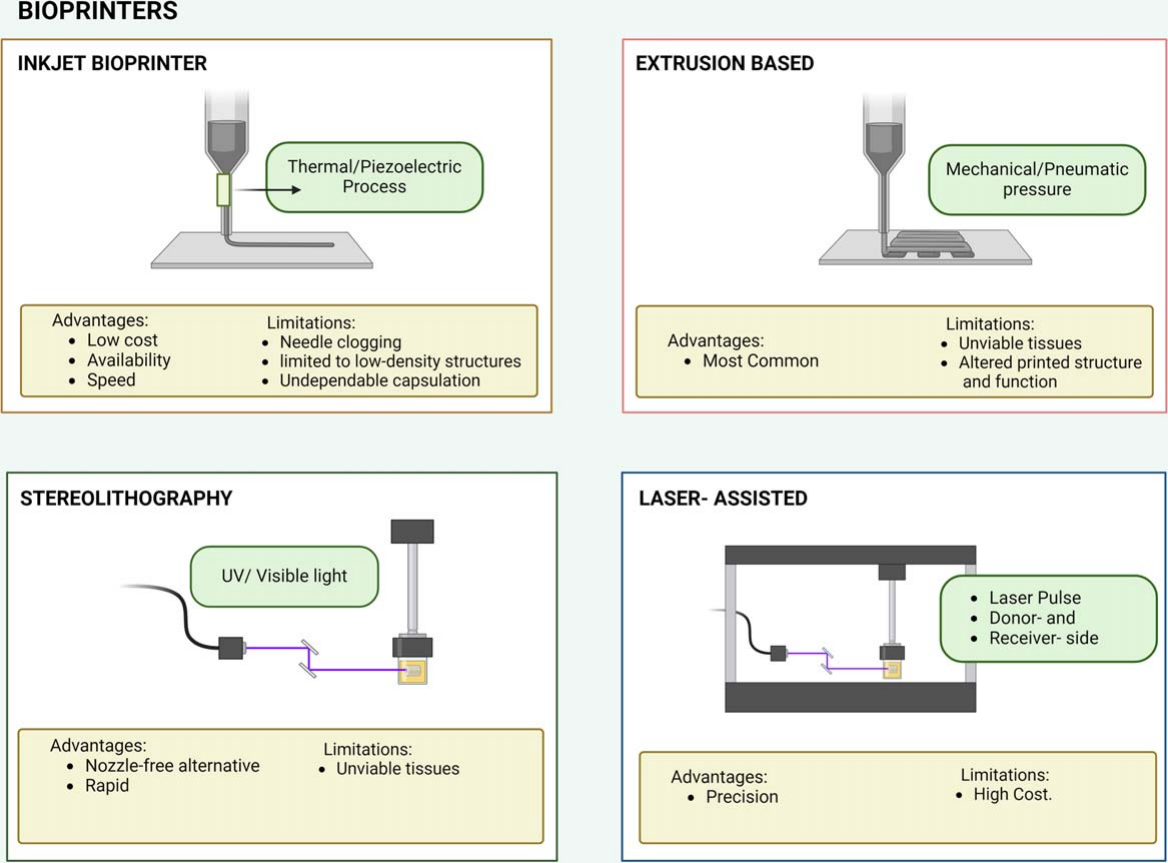
Bioprinting Technologies. This image depicts different bio printing technologies. Inkjet and extrusion use a nozzle-based approach. In contrast, stereolithography and laser-assisted are nozzle-free^5–7^. Extrusion has two categories: pneumatic, which uses compressed air, and mechanical, which uses a motor^5–7^. Both types of extrusion print cell-free-structures/cell-laden with multiple materials, but pneumatic damages cells less and has greater sterility^7^. Inkjet is a droplet-based printer that uses acoustic waves to push material through the nozzle^5–8^. Inkjet is capable of faster printing, higher resolution, and larger build volumes than extrusion-based printers^5–8^. Stereolithography uses photo-crosslinking to create layer-by-layer 3D constructs^6,7^. Once a layer is molded, the substrate on the platform will move down or up to create another layer^6,7^. Laser-assisted transfers cell droplets using laser energy from film to donor surface^6,7^. Both laser-assisted and stereolithography fabricate precise highly-cellular dense 3D constructs^6,7^. Created with BioRender.com.

For bioprinting to be successful and viable, a microarchitecture that is stable and encourages cellular growth is essential^4,6^. As shown in Figures 1 and 2, approaches include nozzle-based methods like inkjet, extrusion, and laser-based methods like laser-assisted bioprinting (LAB) and stereolithography (SLA)^5,6^.

Bioprinters can either create strands or droplets which are arranged in several layers to create a structure^5^. Inkjet-based bioprinting uses a hydrated polymer surrounding the cell as a capsulate in droplet form and permits the pinpoint accuracy of cell position ejected using piezoelectric or thermal processes^5,6,8^. Its low-cost, fast printing speeds, and availability are encouraging. However, limitations to printing only low-density structures, noddle clogging, and undependable capsulation are some of its disadvantages^5,6,8^. Extrusion-based bioprinting is most commonly used as it can utilize mechanical or pneumatic pressure. Limitations include soft tissues with tiny pores making them unviable and the tremendous pressure generated leading to alteration of the printed structure and function^5,6^. SLA utilizes a nozzle-free alternative using ultraviolet or visible light to create layered photosensitive polymers with the assistance of an automatic platform^6^. However, cellular mutations and local cytotoxic effects impair its ability to make viable structures despite its rapid printing speed. LAB requires a laser pulse, both on the donor and receiver side^6^. Although LAB can precisely print multiple types of cells, its high cost limits its effectiveness in LMICs^6^. Table 1 reviews the cost of different printing technologies and compares its advantages and disadvantages.

**Table 1:** Comparison of different bioprinters^4–10^.

Printing technology	Low-cost (USD)	Printing Speed	Resolution	Disadvantage	Advantage
Laser-assisted	~$4000	Medium	>20 μm	Metal particle contamination. High cost. Time consuming	Precise with high resolution. Contact and nozzle-free
Pressure assisted *Extrusion base*	~$2500	Slow	200 μm	Low resolution. Insufficient rigidity. Shape maintained in liquid medium	Print can be molded into various shapes, such as spheroids. Homogenous distribution. Incorporates into cells
Stereolithography *UV-assisted*	~$1500	Fast	10–100 μm	Possible mutations from ultraviolet rays. Free radical damage Strictly print photopolymers. Toxic reagents	Accurate & nozzle-free. Layer-by-layer printing
Inkjet-based	~$320	Fast	20–100 μm	Clogging, mechanical and thermal stress. Strictly liquids	Low-cost and availability. High resolution and fast printing speed. Print organ concentration gradient

With this review, we underline various bioprinting modalities available and discuss how they can be used in LMIC.

## Advances in bioprinting technology for cardiac tissue engineering and regeneration

Bioprinting for CVD has a significant role in surgical planning, optimizing computerized tomography (CT) protocols, medical devices, and training^4,5,9^. Combining bioprinted myocardium and vasculature patches can replace damaged heart tissue with healthy tissue. Additionally, valves with antithrombotic properties can be bioprinted and tailored to all types of patient populations, especially neonates with heart anomalies, who often require cardiac-specific structures, valves, and tissues for survival. Tables 2–4 summarizes cardiac-specific engineering using UV-assisted, inkjet-based, and extrusion-based printing. As we progress towards completing a three-dimensional (3D) cardiac map, we edge closer to the possibility of creating whole biofabricated hearts for transplantation^4,5^.

**Table 2:** Cardiac engineering using UV-assisted techniques^3,4^.

Printing hardware	Cell	Hydrogel	Cofactor	Result
UV-assisted				
Sequential	hiPSC induced cardiac progenitor	None	Signal activator: Wnt	Primary pacemaker induced through WNT5b activating signaling pathway (Wnt) differentiating Nkx2.5+
One head	HEScCM	GelMA	GCaMP	Myocardium mechanical force and calcium transient requires more investigation
	Valvular interstitial cells		None	Heart valve tissue engineered
Two-head	Human coronary artery endothelial cell	MeCol, Alginate, arboxyl functionalized CNTs	None	CNTs temporary improves HCAEC differentiation, proliferation, and lumen-like organization

CNTs, carbon nanotubes; GCaMP, Green fluorescent protein/calmodulin/M13 Peptide; GelMA, methacrylic anhydride gelatine; HEScCM, Human embryonic stem cell-derived CM; hiPSC, human induced pluripotent stem cells; MeCol, collagen methacrylate.

**Table 3:** Cardiac engineering using Inkjet-based techniques^3,4^.

Printing hardware	Cell	Hydrogel	Cofactor	Result
Inkjet-based				
Needle-arrayed	hiPSC-CM	None	Signal activator: Wnt	Cardiac function and angiogenesis
One-head operation	hiPSC-CM and Fibroblast	Gelatin and fibronectin	dECM	Contractility and vascularization

CM, cardiomyocytes; dECM, Decellularized extracellular matrix; hiPSC, human induced pluripotent stem cells; HUVEC, Human umbilical vein endothelial cell.

**Table 4:** Cardiac engineering using extrusion-based techniques^3,4^.

Printing hardware	Cell	Hydrogel	Cofactor	Application	Result
Extrusion-based					
Rapid digital light process	HiPSC-CM	GelMA	Porcine LV dECM	Mix cell into hydrogel to form micro- structure resembling stripe in vitro	Complex geometry myocardial microstructure with resolution 30 μm
Visible light assisted	Cardiac fibroblast and iPSC-CM	Furfuryl-gelatin and fibrin	None	To study the combination of cardiac fibroblast and cardiomyocyte	Suggest connexin-43 as important communication
	Human neonatal cardiac progenitor cell	GelMA	Cardiac dECM	Myocardial patch test in vitro and in vivo placed on the heart of healthy rat	After 14 days, microangiogenesis and successful attachment
One head	hiPSC	MeCol, GelMA, laminin 111, fibronectin	None	Differentiated into cardiomyocytes for vitro testing	Secondary heart structures can be printed with contractile properties
Two-heads	IPS-CM	Fibrin	None	Biological evaluation	Myocardial-like structure and functions
	Human EC and CM	Collagen composited FRESH	VEGF	Heart model successfully created own vascularization system	Possibilities FRESH v2.0 can engineer multiplex scaffolds
	Endothelial cells and hiPS-CM	Gelatin	Porcine omentum decellularized	One step a thick vascularized heart patch and heart printed	Possibly suitable for clinical use
	HUVEC and H9c2	PVA, agarose, and alginate	Platelet rich plasma	To study heart orientation properties in vitro	Cell-hydrogel ratio can engineer whole heart
Three-heads	Rat ventricular primary CM	PCL, fibrinogen, hyaluronic acid, gelatin, glycerol, aprotinin	None	Testing biomechanics and electrophysiology of cardiac patch	Creating functional heart requires further micro-sensing
Multiheads	Cardiac fibroblast and CM	Fibronectin and GelMA	None	Printing process assessed to test mechanical forces and biological activity	Lowing GelMA, add extra molecules, and two-cell bioink may improve network formation and cell survival
	CM-LV of neonatal rat	PEVA and type 1 atelo-collagen	Porcine LV dECM	Added living cells to scaffold	CM different response to culture conditions compared to bioink composition
	HUVEC and Mice IPS-CM	PEG-fibrinogen and alginate	None	Printed layers of scaffold to evaluated organization in vivo and vitro	Tissue can merge with host heart

FRESH, freeform reversible embedding of suspended hydrogels; PEVA, polyethylene-vinyl acetate; VEGF, vascular endothelial growth factor.

### Whole biofabricated hearts

Creating a biofabricated heart involves a patient-specific CT model which can be used as a 3D map. Extraction of dermal fibroblasts from a patient’s skin can be induced to pluripotent stem cells (iPSC) with transcription factors Klf4, Sox2, OCT 3/4, and c-Myc. Once the iPSC population is formed, cardiac-specific lineages can differentiate to form cardiomyocytes, endothelial cells, and other cell populations^5^. Following printing, the heart needs to be cultured, conditioned, and matured in a bioreactor for several days. Testing physiological parameters must be completed before implantation. A future direction in biofabricated heart transplantation is to embed sensors within the scaffolding to record several cardiac-specific parameters. Cells are immune-tolerant which means that immunosuppression therapy is not required^5^.

### Valve replacement

The early stages of valve replication focuses on biological, structural, and physicochemical properties. A scaffold of an aortic valve using photocrosslink-printing can attain a 10-fold increase in elastic content and shape fidelity^4^. The use of computer-assisted design and computer-assisted manufacturing can replicate accurate valve structures with cells derived from valves and bioink biomaterial^5^. For a fully biofabricated heart, valves must be bioprinted separately, having an antithrombotic lining on both surfaces and be deployed robotically into their correct anatomical location when the heart is nearing completion of its reconstruction^5^. Recently, a 3D-printed SLA model was used to determine the exact cardiac anatomy of a patient who required an aortic valve replacement with a significant history of right and left descending coronary artery bypass grafting (CABG) that created anatomical and surgical challenges^11^.

### Myocardium heart patches

Myocardium patches using a laser induced forward transfer based printing technique was able to preserve myocardial function at and around the site of injury following an infarction by enhancing angiogenesis. These were created using endothelial cells and mesenchymal stem cells laden with polyester urethane urea. Additional techniques using extrusion-printing with human-derived cardiomyocyte progenitor cells (hCMPCs) and hyaluronic acid/gelatin or alginate bioinks implanted in mouse myocardial infarction (MI) models improved cardiac function and survival for 1 month^4^. The implantation of decellularized extracellular matrices (dECM) bioink tissue with mesenchymal stem cells/vascular endothelial growth factors (VEGF) and cardiac progenitor cells promoted prolonged cell survival, vascularization, and tissue remodeling at the myocardial injury site^4^. A cardiac patch ‘free of biomaterial’ using iPSC, including cardiomyocytes, endothelial cells, and fibroblasts, exhibited spontaneous beating, synchronized electric conduction, and ventricular waveform action potential. Implantation of the biomaterial free cardiac patch in vivo with native rat myocardium led to successful vascularization^4^.

### Microcirculation and coronary arteries

Dysfunction of the cardiac microcirculation leads to ischemic heart disease, a recognized component of congestive heart failure^5^. A significant obstacle to 3D printing has been creating a functional microcirculation to perfuse printed tissues^5^. An alternative is placing a 3D cardiac patch on the epicardial surface of the ischemic myocardium^4,5^. The patch could equilibrate the dysfunction and prevent complications of congestive heart failure^4,5^. A breakthrough using intact microvascular fragments combined with fat vascular cells has created attainable microvascular constructs^5^. Unfortunately, size limitations of 3D printers prevent printing of microvascular structures such as venules and arterioles.

The lack of maturation of endothelium on the luminal surface is the reason for early thrombosis following CABG^5^. To avoid early luminal thrombosis when bioprinting vessels, the lumen must possess antithrombotic properties with rapid endothelialization promoting neovascularization^4,5^. Autologous endothelial lining was created on a synthetic graph called endothelialization in vitro which takes 6–8 weeks to mature prior to implantation^5^. Preclinical models and various clinical trials using autologous endothelial cells to nurture graphs are promising approaches^5^.

Bioprinting perfusable vasculature faces challenges in multilayer printing of the tunica adventitia, tunica media, and intima^4^. The ability to create a 3D spheroid is a breakthrough in bioprinting of small diameter structures. Additional attempts have led to the use of modified printer heads to create large vessel conduits in tubes of different sizes^5^. Further research and development is required to advance tissue engineering and overcome these challenges^4,5^.

## Applications and possibilities in low-income countries or middle-income countries (LMIC)

**Figure 3:**
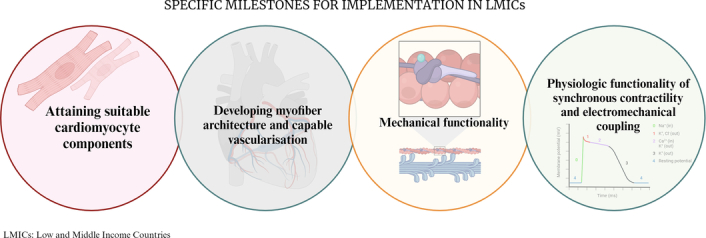
Implementation milestones. This figure depicts the four specific milestones for implementation in LMICs. Created with BioRender.com.

CVD affects high-income countries differently than LMICs. High-income countries are affected by CVD 10–15 years after LMICs. The burden of CVD on LMICs affects economic growth by reducing the number of employees in the workforce^12^. The potentially productive years of life lost is ~9.2 million (3572 per 100 000) in India and 6.7 million (1595 per 100 000) in China affecting a demographic within 35–64 years of age which is expected to rise^12^. The significant disparities in access to healthcare between rich and developing countries have led the WHO to create a Clinical Procedures Unit for LMICs. The Clinical Procedures Unit aims to provide essential anesthetic and surgical services in LMICs through the Emergency and Essential Surgical Care Project^13^. Currently, 3D printing prototypes in LMICs are used in developing basic medical supplies such as finger splints, crutches, umbilical clamps, and low-cost prosthetic limbs with controllable fingers^13^. The application of biofabricated hearts in LMICs must first reach specific milestones, as shown in Figure 3: (1) cardiomyocyte components with suitable cells and biomaterial, (2) vascularization capable of perfusion, (3) mechanical, and (4) physiologic functionality of synchronous contractions and electromechanical coupling^4^. With the ability of biofabrication to create patches for strengthening the left ventricle in case of failure and/or MI, millions of lives can be positively affected, especially those in LMICs^5,13^. Addressing the availability challenges of donors and augmenting resources is a viable solution, but it comes with a significant drawback – any importation of materials will only escalate costs. The optimal approach appears to be a gradual, phased expansion of infrastructure and the cultivation of in-house capabilities for bioprinting. While the potential is immense and the future appears promising, achieving a better tomorrow relies on meticulous planning today.

## Unprecedented biomimicry compared to autografts (potential to provide revascularization of the scaffolds and microfluidic devices)

The shortage of heart donors has permitted the use of marginal organ donors^14^. In 2015, the International Society for Heart and Lung Transplantation (ISHLT) Registry reported 5074 heart transplants with a median survival of 10.7 years in the adult population and 16.1 years in the pediatric population across 285 centers^14^. Significant complications occur within the first month after surgery^15^. The leading cause of death within 30 days of a heart transplant is graft failure whereas the most common long-term complication is cardiac allograft vasculopathy^14,16^. Homogeneous cell-laden scaffolds have a low risk of rejection and are rapid in integrating with the native organs^6^. Bioprinting cardiac tissue requires the cell source to be of autologous origin, which can be easily differentiated to the preferred cell type, maintain cellular functionality once differentiated, and be nonantigenic^4^. The cellular population of the heart consists of smooth muscle cells (SMCs), cardiac fibroblasts (FBs), cardiomyocytes (CMs), endothelial cells (ECs), and conduction cells (Purkinje and pacemaker)^4^. Embryonic stem cells (ESCs), adult stem cells, and iPSCs are widely used to differentiate into cardiac cell types^4^. Mesenchymal stem cells (MSCs) cannot differentiate into cardiac tissue but can differentiate into the supportive cardiomyocyte-like lineage, limiting their use in cardiac tissue models^4^. The use of ESCs has greatly improved cardiac functionality, thus increasing survival post-MI, but limitations continue to restrict their use^4^. Growth factors can stimulate cardiac stem cells (CSCs) to induce myocardium-supportive cell lineages like vascular endothelial, smooth muscle, and myocyte cells. Supporting evidence has shown the heart has self-renewal potential, and the use of CSCs can eliminate the risk of immunologic rejection^4^. Induced pluripotent stem cells, however, can be reprogrammed to induce into the entire cellular population of the cardiovascular system in vitro^4^. Using iPSCs for scaffolding avoids ethical and immunological rejection^4^.

**Figure 4:**
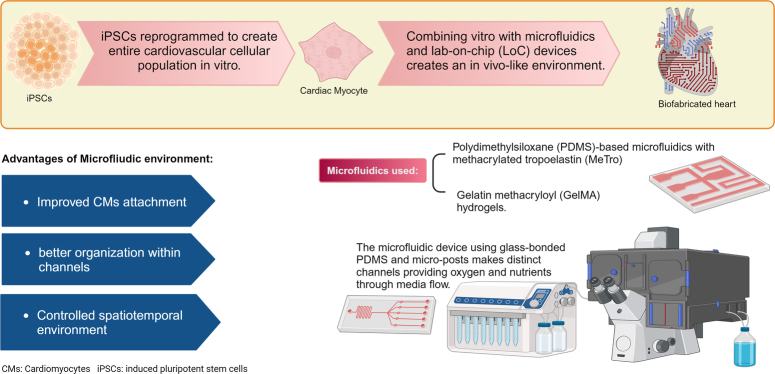
Microfluidics in bioprinting. The microfluidic device using glass-bonded PDMS and micro-posts makes distinct channels providing oxygen and nutrients through media flow. CMs, cardiomyocytes; iPSCs, induced pluripotent stem cells. Created with BioRender.com.

Combining in vitro microfluidics, (Figure 4) and lab-on-chip (LoC) devices creates an in vivo-like environment^17^. Polydimethylsiloxane (PDMS)-based microfluidics with methacrylated tropoelastin (MeTro) and gelatin methacryloyl (GelMA) hydrogels improved CMs attachment and organization within channels^17^. The MeTro coating appeared to have better attachment and beating when analyzed from neonatal rat CMs culture^17^. Static cell cultures’ physical and chemical limitations create barriers leading to uncontrolled sheer stress, altering cell morphology, focal adhesions, and transformation^17^. By controlling the spatiotemporal environment through a microfluidic environment, mechanical myocardium cyclic uniaxial contraction and electrochemical stimulation can be achieved^17^. Mimicking the microvasculature through culturing endothelial cells, the microfluidic device using glass-bonded PDMS and micro-posts created three separate channels which provided oxygen and nutrients through media flow^17^. Integrating 3D-bioprinted fabrication and microfluidic chips can create a realistic approach to cardiac disease^17^.

## Current developments of 3D-bioprinting and future perspectives

Leveraging 3D bioprinting has the potential to revolutionize patient-specific care. Presently, 3D bioprinting is actively employed in creating surgical, tumor, and disease models. However, the demand for additional cadavers has restricted progress in enhancing preoperative preparations for surgeries. With the aid of a CT scanner, it becomes possible to generate a patient-specific model of an organ, complete with vessels, ducts, specific organ contours, and tumors. This innovative approach opens new avenues for precision and customization in medical procedures. A life-size model of a liver was printed for less than $150, which aided in a successful laparoscopic right hemihepatectomy^18^. For less than $100, ureter, kidney, and renal pelvis models were printed to perfect pediatric laparoscopic pyeloplasty. More recently, congenital heart disease models have been printed to gain in-depth knowledge of the intricate anatomy and patient-specific surgical strategies^18^. To replace injured vessels proceeding with surgical reconstruction, a hybrid 3D scaffold with pericardium and polyethylene glycol was developed. The hybrid 3D scaffold decreased macrophage signaling, reducing the inflammatory process to enhance vascular graft healing^18^. 3D-printed prostheses and implants has created the ability to develop individualized therapy. The low-cost of 3D bioprinting has supported children with upper-extremity reduction. Recent studies have shown that a 3D printed hand improved quality of life, and children could participate in daily activities^18^. The future of 3D bioprinting for CVD promises groundbreaking advancements. Anticipated developments include the creation of patient-specific, functional cardiovascular organs and addressing transplant challenges. Customized vascular grafts, produced with intricate precision, hold the potential for improved outcomes in vascular disease patients. Bioprinted cardiac tissues are poised to revolutionize drug testing and personalized medicine, providing a platform for studying individualized drug responses – adding a new phase to the current structure of clinical trials. Tables 5–7 summarizes additional advancements in tissue engineering. Integrated electronic components could enhance functionality, enabling biohybrid systems with monitoring capabilities, and integrated pacemakers. Bioprinted cardiac patches in the future may also stimulate tissue regeneration after cardiovascular events. Bioprinted vascular stents can be another breakthrough, which can redefine interventional cardiac care. Ethical considerations will play a pivotal role in ensuring responsible advancements, as the convergence of bioprinting with other technologies reshapes the landscape of cardiovascular care.

**Table 5:** Further application of inject-based engineering^6^.

Study	Cell	Biomaterial	Results	Significance
Inkjet-based				
Vivo	NHDFEK, DMEC	Fibrinogen, collagen, thrombin	After 6-week scaffold demonstrated 17% improved wound contraction	Bioprinting grafts superior to commercial keratinocyte/fibroblast grafts
Vivo	HMECs	Fibrin	After 21 printed tubular structure	Promising Microvascular tissue printing
Situ	MSCs and AFS	Collagen-Fibrinogen	Angiogenesis in subcutaneous adipose tissue when used on mice backs to repair full thickness wounds	Revascularization in tissue and quick closure of full thickness burns
Situ	Human chondrocytes	PEGDMA	Enhanced tissue integration when printed on defective femoral condyles	Direct in situ printing can enhance integration
Vitro	CM (feline adult); cardiac muscle cell (HL1)	Alginate	In large scaffold cells survived and pulsated with electric stimulation	Viable myogenic tissue printed
Vitro	Neuronal NG108, Schwann cells, neuronal	Cell suspension	Lengthening of neurite and high cell viabilities	Neuronal network in large tissue prior to implantation

AFS, amniotic fluid-derived stem cells; CM, cardiomyocytes; DMEC, dermal microvascular endothelial cells; HMECs, human microvascular endothelial cells; MSCs, mesenchymal stem cells; NHDFEK, neonatal human dermal fibroblast and epidermal keratinocytes; PEGDMA, Poly(ethylene glycol) dimethacrylate.

**Table 6:** Further application of Laser-assisted engineering^6^.

Study	Cell	Biomaterial	Results	Significance
Laser-assisted				
Vivo	Human keratinocytes, mouse fibroblast	Collagen	After 11 days in subcutaneous tissue of nude mice, vascularization and multiple layers of epidermis	Skin graft required to utilize
Situ	MSCs	nHA	Pulsating blood vessels	Printing in mouse calvaria produces scaffold
Vitro	HUVECs	Human Osseous Cell sheets	Tubule-like structure after culturing for 21 days	Sheets of soft tissue

HOCS, human Osseous Cell sheets; HUVECs, human umbilical vein endothelial cells; nHA, nanocrystalline hydroxyapatite.

**Table 7:** Further application of extrusion-based engineering^6^.

Study	Cell	Biomaterial	Results	Significance
Extrusion-based				
Situ	Bone marrow-derived mesenchymal stem cells	HA-GelMA	Directly cultured cells into defective sheep cartilage	Cartilage reconstructed
Vitro	HUVECs	GelMA	Printed endothelium Lumen-like structure. After 7–10 days contracted with rate of 60 bpm	Successful 3D myocardium-endothelialized-on a chip
Vitro	Human chondrocytes	Alginate PLA fibers	High cell viability	Hydrogen mechanical properties improve with PLA fibers
Vitro	Chondrocytes	Collagen sodium alginate/sodium alginate/ agarose	Increase cell culture scaffold and cell adhesion	Potential for cartilage regeneration

GelMA, methacrylic anhydride gelatine; HUVECs, human umbilical vein endothelial cells.

## Clinical challenges and considerations

3D bioprinting has been an emerging field making huge advancements over the past decade. In the early stages, the biggest hurdles were focused on printing viable cells that can be accepted by a patient’s body. Creating a simple organ such as a bladder outside of the body was the first major accomplishment; leading to today’s challenges of complex organ development^19^. These advancements are due to a better understanding of both stem cells and tissue structure. Currently, the next phase of 3D bioprinting is focused on more complex organs such as skin, bone, eyes, and vascular structures. The largest hurdles at this time stem from maintaining tissue viability during the printing process, before implantation into a living host. Highly vascular structures require blood and nutrients supplied within a short amount of time before tissue decay is noted. One of the biggest factors inhibiting cheap and viable organ transplants is the speed it takes to print an organ, followed by making it available to a surgeon to implant it^19^. Engineers are constantly trying to overcome these challenges by developing quicker printing methodologies and utilizing other forms of building blocks. One of the newest advancements is the utilization of sugar to create vascular stents^20^. With the developments in computational biology – the future of cardiac care seems bright^21,22^ – the day is not far when these 3D models will be the scaffold for not only directing cardiac interventions but also ‘printing’ the needed tissues. Artificial Intelligence, nanotechnology and 3D bioprinting have enhanced the treatment capabilities for infectious diseases and have allowed us to go beyond animal-based solutions, it is high time we utilize the growing technology to assist and advance cardiovascular care^23–26^. The current landscape of 3D bioprinting is far from low-cost organ transplantation, however, over the next few years with continued research and advancements lower cost transplants may become a real possibility.

## Limitations and complications of bioprinting

The evolution of 3D bioprinting has introduced a paradigm shift in the management and treatment of diverse CVD. The aforementioned domains have elucidated strategies in additive manufacturing (AM) while concurrently exposing inherent limitations. A conspicuous impediment resides in the transition from static 3D printing to dynamic 3D bioprinting for the synthesis of cohesive transplantable organs. The segmentation of individual structures for subsequent assembly engenders notable challenges. Postsuccessful biofabrication assembly, endeavors in anastomosis encounter substantial hurdles. Before clinical viability, rigorous longitudinal studies with considerable sample sizes are imperative for mid-to-long-term validation of 3D bioprinted organs. Financial constraints, stemming from elevated costs, obfuscate the feasibility of this modality as a treatment option but compared against unavailability and need based costs – the benefits surely presents a worthy alternative to human donated organs/animal-based options. Ethical apprehensions regarding the selection of suitable stem cells for bioink, and concerns to the structural, mechanical, and biocompatible facets, are ongoing difficulties. Despite these challenges, a meticulous refinement of the structural components in 3D bioprinted hearts holds promise for diverse applications, encompassing tissues for surgeries, vessels, ventricles, valves, and patches. Effectively transforming encapsulated bioink into scaffolds faces challenges such as cytotoxicity, necrosis, sheer stress, mutations, and resolution discrepancy. Combining different fabrication techniques can bioprint primary scaffolds and secondary supporting structures to create a proficient and feasible manufacturing process. The combination of LAB and inkjet printing can accurately position cells; however, inkjet inefficiently creates 3D architecture, whereas LAB prefabricates scaffolds at a higher cost. Extrusion bioprinting can print large 3D structures, but cell death ensues. Finding the optimal combination of bioprinting methods is key to generate fully functional tissues and organs.

## Conclusions

The intricate nature of cardiac structures poses a challenge to the 3D bioprinting of tissues, necessitating the separate printing, assembly, and transplantation of individual components. To address this, various 3D mapping approaches have been explored, enabling the bioprinting of specific components tailored for disease-specific treatments. Subsequent advancements in this mapping strategy have yielded components such as valves, coronary vessels, ventricles, and tissues. This ongoing progress has introduced critical checkpoints and novel applications of cell-specific bioprinting and LoC systems. The developments have indeed given a new ray of hope allowing cost-effective solutions for organ/tissue replacements, especially for LMICs. However, it is imperative to acknowledge certain limitations in the current landscape of 3D bioprinting.

## Ethical approval

Not applicable.

## Consent

Not applicable.

## Sources of funding

This research did not receive any specific grant from funding agencies in the public, commercial, or not-for-profit sectors.

## Author contribution

A.M., A.M., D.F., and P.K.: writing – original draft and writing – review and editing; I.P.: conceptualization, writing – original draft, and writing – review and editing; A.M.M.: writing – original draft; T.R.: writing – original draft and writing – review and editing; Y.S.: conceptualization, writing – review and editing; T.B.E.: data collection, revision, and supervision.

## Conflicts of interest disclosure

The authors have no conflict of interest.

## Research registration unique identifying number (UIN)

1. Name of the registry: not applicable.
2. Unique identifying number or registration ID: not applicable.
3. Hyperlink to your specific registration (must be publicly accessible and will be checked): not applicable.

## Guarantor

Talha Bin Emran.

## Data availability statement

The data in this correspondence article is not sensitive in nature and is accessible in the public domain. The data is therefore available and not of a confidential nature.

## Provenance and peer review

Not commissioned, internally peer-reviewed.
